# Effect of Antioxidant Supplementation on Exercise-Induced Cardiac Troponin Release in Cyclists: A Randomized Trial

**DOI:** 10.1371/journal.pone.0079280

**Published:** 2013-11-19

**Authors:** Lieke J. J. Klinkenberg, Peter T. Res, Guido R. Haenen, Aalt Bast, Luc J. C. van Loon, Marja P. van Dieijen-Visser, Steven J.R. Meex

**Affiliations:** 1 Department of Clinical Chemistry, Cardiovascular Research Institute Maastricht (CARIM), Maastricht University Medical Center (MUMC), Maastricht, The Netherlands; 2 Department of Human Movement Sciences, School for Nutrition, Toxicology and Metabolism (NUTRIM), Maastricht University Medical Center (MUMC), Maastricht, The Netherlands; 3 Department of Toxicology, School for Nutrition, Toxicology and Metabolism (NUTRIM), Maastricht University Medical Center (MUMC), Maastricht, The Netherlands; Postgraduate Medical Institute & Hull York Medical School, University of Hull, United Kingdom

## Abstract

**Background:**

Cardiac troponin is the biochemical gold standard to diagnose acute myocardial infarction. Interestingly however, elevated cardiac troponin concentrations are also frequently observed during and after endurance-type exercise. Oxidative stress associated with prolonged exercise has been proposed to contribute to cardiac troponin release. Therefore, the aim of this study was to assess the effect of 4 week astaxanthin supplementation (a potent cartenoid antioxidant) on antioxidant capacity and exercise-induced cardiac troponin release in cyclists.

**Methods:**

Thirty-two well-trained male cyclists (age 25±5, weight 73±7 kg, maximum O_2_ uptake 60±5 mL·kg^−1^·min^−1^, W_max_ 5.4±0.5 W·kg^−1^; mean ± SD) were repeatedly subjected to a laboratory based standardized exercise protocol before and after 4 weeks of astaxanthin (20 mg/day), or placebo supplementation in a double-blind randomized manner. Blood samples were obtained at baseline, at 60 min of cycling and immediately post-exercise (≈ 120 min).

**Results:**

The pre-supplementation cycling trial induced a significant rise of median cardiac troponin T concentrations from 3.2 (IQR 3.0–4.2) to 4.7 ng/L (IQR 3.7–6.7), immediately post-exercise (p<0.001). Four weeks of astaxanthin supplementation significantly increased mean basal plasma astaxanthin concentrations from non-detectable values to 175±86 µg·kg^−1^. However, daily astaxanthin supplementation had no effect on exercise-induced cardiac troponin T release (p = 0.24), as measured by the incremental area under the curve. Furthermore, the elevation in basal plasma astaxanthin concentrations was not reflected in changes in antioxidant capacity markers (trolox equivalent antioxidant capacity, uric acid, and malondialdehyde). Markers of inflammation (high-sensitivity C-reactive protein) and exercise-induced skeletal muscle damage (creatine kinase) were equally unaffected by astaxanthin supplementation.

**Conclusion:**

Despite substantial increases in plasma astaxanthin concentrations, astaxanthin supplementation did not improve antioxidant capacity in well-trained cyclists. Accordingly, exercise-induced cardiac troponin T concentrations were not affected by astaxanthin supplementation.

**Trial registration:**

ClinicalTrials.gov NCT01241877

## Introduction

Myocardial necrosis is accompanied by the release of cardiac troponin (cTn) into the bloodstream, which makes cTn the preferred biomarker for the diagnosis of acute myocardial infarction (AMI) [1]. Interestingly, elevated cTn concentrations are not restricted to AMI, but are also observed in a wide range of cardiac and non-cardiac pathologies e.g. renal failure, pulmonary embolism, myocarditis and congestive heart failure (an extensive list has been published in [2], [3]). In all these pathological settings elevated cTn concentrations are consistently associated with poor prognosis [4]–[7]. An intriguing example of non-AMI related cTn release in healthy subjects occurs after moderate and highly intensive endurance exercise such as marathons, cycling events and long-distance walking [8]–[12]. cTn levels in blood rise quickly during exercise, and unlike AMI, they return quickly to normal in approximately 24–48 hours [9], [13], [14]. To date it has not been clarified whether exercise-induced cTn release is a physiologic or pathologic phenomenon. There is no epidemiological evidence thus far that links the magnitude of exercise-induced cTn release to poor prognosis. The faster kinetics of exercise-induced cTn release, and the possible presence of a cytosolic unbound cTn fraction in the cardiomyocyte [15], triggered the postulation of a distinct release model for cTn during exercise, unrelated to myocardial cell death [16]. This hypothetical model states that exercise-induced cTn release could be attributed to increased cardiomyocyte permeability (through mechanical stress or increased production of oxidative radicals) and subsequent early troponin release from a cytosolic cTn pool, possibly through membrane blebbing [16], [17]. This atypical model of benign cTn release was coined the “reversible injury hypothesis”. It challenges the dogma that increased cTn concentrations always reflect cell death, and unites two apparently incompatible observations: 1) the frequent occurrence of exercise-induced cTn release [18], and 2) the limited cardiomyocyte regenerative capacity [19]. This hypothesis however, has been opposed by a number of observations that warrant prudence before classifying all exercise-induced cTn rises as benign. First, the most pronounced post-exercise cTn concentrations have been observed in the least trained subjects [20]. Second, baseline cTn levels, which are robust predictors of cardiovascular morbidity and mortality in virtually every patient group investigated, explained up to 80% of the cTn variation after exercise [21]. Finally, a strong correlation has been observed between post-exercise cTn concentrations and short-term right ventricular functional impairment [22]. Over time, repetitive strain on the right ventricle may lead to chronic structural changes, as demonstrated by a high prevalence of chronic structural changes in athletes with the highest cumulative exposure to endurance competition [23]–[25].

The identification of the biological mechanism that underlies exercise-induced cTn release is an active and relevant topic of discussion [17]. Oxidative stress associated with prolonged exercise has been proposed to contribute to cTn release [26], [27]. If true, supporting endogenous defense systems with additional doses of antioxidants could therefore be an important strategy to examine the role of oxidative stress as a mechanism for exercise-induced cTn release. Therefore, we subjected male trained cyclists to a laboratory based standardized exercise protocol before and after 4 weeks of astaxanthin (a potent cartenoid antioxidant) or placebo supplementation. We hypothesized that astaxanthin supplementation attenuates oxidative stress, inflammation and muscle damage following cycling exercise and hence reduces exercise-induced cardiac troponin release.

## Materials and Methods

### Ethics Statement

This study complies with the Declaration of Helsinki and was approved by the Institutional Review Board and Ethics Committee at Maastricht University Medical Center. All participants gave written informed consent. This study was registered at clinicaltrials.gov as NCT01241877. The protocol for this trial and supporting CONSORT checklist are available as supporting information; see Checklist S1 and Protocol S1.

### Trial Design

The study was designed as a double-blind, placebo-controlled randomized parallel group trial. Thirty-two healthy male volunteers were recruited from 01-12-2010 till 01-06-2011 via local cycling clubs. We included healthy, young (age between 18 and 30 years), endurance-trained male cyclists, with a training history of more than 3 sessions per week for more than one year. The study was conducted between 01-01-2011 and 01-11-2011 at the Department of Human Movement Sciences at Maastricht University Medical Center. All subjects were competitive cyclists or triathletes who had been involved in regular cycling training (more than 3 sessions per week with 11±5 hours per week) for at least two years.

### Study Protocol

The study was designed to examine the effect of 4 weeks of astaxanthin supplementation on exercise-induced cTn release. This study is part of a greater randomized trial examining the impact of astaxanthin supplementation on muscle metabolism and exercise performance, as primary outcome measures [28]. Briefly, subjects visited our laboratory on four occasions, two of which were scheduled prior to the start of the main experimental period. The first visit consisted of an incremental cycling exercise test to exhaustion to determine subjects’ maximal workload capacity (W_max_) and maximum oxygen uptake (VO_2 max_). The second visit consisted of a familiarization test that was identical in design as the main experimental test days. The actual experimental period (visit 3 and 4) consisted of two identical standardized cycling trials, separated by a 4 week supplementation period with astaxanthin or placebo. The pre- and post-supplementation trials were performed on the same time and day of the week for each subject. On each test day, subjects reported to the laboratory at 08.00 after an overnight fast. A Teflon catheter (Baxter, Utrecht, the Netherlands) was inserted into an antecubital vein for blood sampling. The exercise protocol consisted of 60 min steady-state moderate intensity exercise (50% W_max_) followed by a validated time-trial [29], in which athletes performed a set amount of work at maximal performance for about ≈ 60 min. Blood samples were obtained at baseline, at 60 min of cycling and immediately post-exercise (≈ 120 min).

### Supplementation Protocol

Participants were randomly assigned in a double-blind fashion to receive either 20 mg/day of astaxanthin or placebo for 4 weeks. Randomization was performed by a random number generator (www.random.org). Astaxanthin is a xanthophyll carotenoid pigment with potent antioxidative and anti-inflammatory capacities and has previously been shown to attenuate exercise-induced oxidative damage in mouse skeletal and cardiac muscle [30], [31]. The astaxanthin supplementation consisted of a natural astaxanthin extract from the microalga *Haematococcus pluvialis* (80 mg of 5% astaxanthin) dissolved in sun flower oil in gelatin capsules with added vitamin C (60 mg per capsule) and vitamin E (10 mg per capsule) (BioReal, Gustavsberg, Sweden). Participants ingested 2 capsules (4 mg astaxanthin/capsule) with breakfast and 3 capsules with dinner throughout the 4-week intervention period. The placebo supplement was externally identical to the astaxanthin capsule, without the phytonutrient content. The placebo group followed the same supplementation protocol as the astaxanthin group. The supplementation was assigned by an independent investigator, so that both the subjects and investigators were blinded to the treatment. Supplementation compliance was assessed by the number of pills that were left over in the pill bottle after the supplementation period.

### Physical Activity and Dietary Standardization

During the study, subjects were allowed to consume their normal diets but were instructed to refrain from astaxanthin-rich foods such as salmon, lobster, and shrimp. All subjects were also instructed to refrain from physical exercise for two days before each test day. In addition, we asked subjects to record their food intake for two days before the pre-supplementation test day and to replicate their food intake two days before the post-supplementation test day. All subjects received a standardized dinner the evening prior to each experimental test day (62±4 kJ·kg bodyweight^−1^, consisting of 50 energy percent (En%) carbohydrate, 10 En% protein, and 40 En% fat).

### Biochemical Analyses

Plasma and serum samples were stored at −80°C until analysis. Serum cardiac troponin T (cTnT) was measured using the high-sensitivity cTnT assay (Roche Diagnostics) on the Elecsys 2010 using lot no. 167650. The measuring range of this assay is 3–10,000 ng/L, with a 99^th^ percentile in apparently healthy individuals at 14 ng/L, which is clinically employed to diagnose AMI [32]. Serum creatine kinase (CK) was measured on Roche Cobas 6000 analyzer. Serum high-sensitivity C-reactive protein (hsCRP) was measured on the BN Prospec (Siemens). Plasma carotenoid concentrations and antioxidant markers (trolox equivalent antioxidant capacity (TEAC), uric acid, and malondialdehyde (MDA)) have been measured in the setting of a previous study and reported accordingly [28].

### Statistical Analysis

To be able to detect a 10% reduction in exercise-induced cTn release (from 7.0±1.4 to 6.3±1.3 ng/L) due to astaxanthin, with a two-sided significance level of 5% and 80% power, a total of 32 subjects were recruited. No intervention has been reported that effective reduces exercise-induced cTn release. The effect size of 10% was therefore derived from the antioxidant-induced reduction of skeletal muscle damage after exercise [33], [34]. In these studies 20–30% reductions of CK levels were obtained through antioxidant supplementation. Assuming a similar mechanism and effect size in cardiac muscle, we felt that a <10% reduction would exclude a major role for antioxidants in this setting. The Kolmogorov-Smirnov test was used to evaluate the normality of the data. Participant characteristics are presented as the mean ± SD, biochemical parameters are shown as median and interquartile range. Paired data was tested using the Mann Whitney U test, non-paired data via the Wilcoxon Signed-Rank test. The incremental area under the curve (iAUC) was used as a summary measure for exercise-induced cTnT release. The effect of antioxidant supplementation was assessed by analysis of variance (ANOVA), using the iAUC at pre-supplementation as covariate. Statistical analysis was performed using SPSS, version 18.0.

## Results

### Participants Characteristics

**Figure 1:**
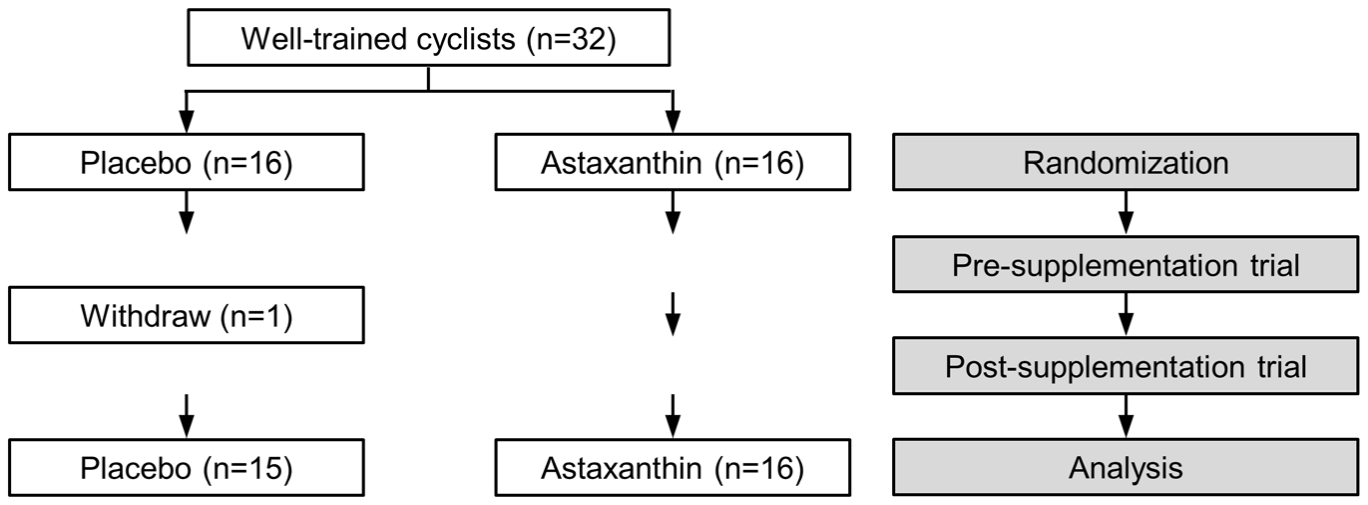
Flow chart of the trial.

A detailed flow of the trial is described in Figure 1. Thirty-two well-trained cyclists were included in the study. One subject in the placebo group left the study prematurely for medical reasons, unrelated to the study protocol. Subjects’ baseline characteristics are shown in Table 1. No differences in age, weight, maximum O_2_ uptake, maximum workload capacity or the average hours of training per week were observed between the placebo (n = 15) and astaxanthin group (n = 16).

**Table 1:** Participants characteristics.

	All participants	Placebo	Antioxidant
	n = 31	n = 15	n = 16
Age (years)	24.7±4.7	25.0±5.5	24.5±3.9
Weight (kg)	72.9±7.2	73.0±8.5	72.9±6.1
Maximum O_2_ uptake (mL·kg^−1^·min^−1^)	60.2±4.8	59.5±5.4	60.9±4.3
Maximum workload (W·kg^−1^)	5.4±0.5	5.3±0.5	5.5±0.5
Training (hours/week)	10.9±4.2	10.5±4.9	11.2±3.5

Data are presented as mean ± SD.

### Astaxanthin Supplementation

In the placebo group, plasma astaxanthin concentrations were undetectable throughout the study. In the astaxanthin group, plasma astaxanthin concentrations were significantly increased from non-detectable values to 175±86 µg·kg^−1^. There were no differences in other cartenoid intake within or between groups during the study.

### Astaxanthin does not Reduce the Magnitude of Exercise-induced Cardiac Troponin Release

**Figure 2:**
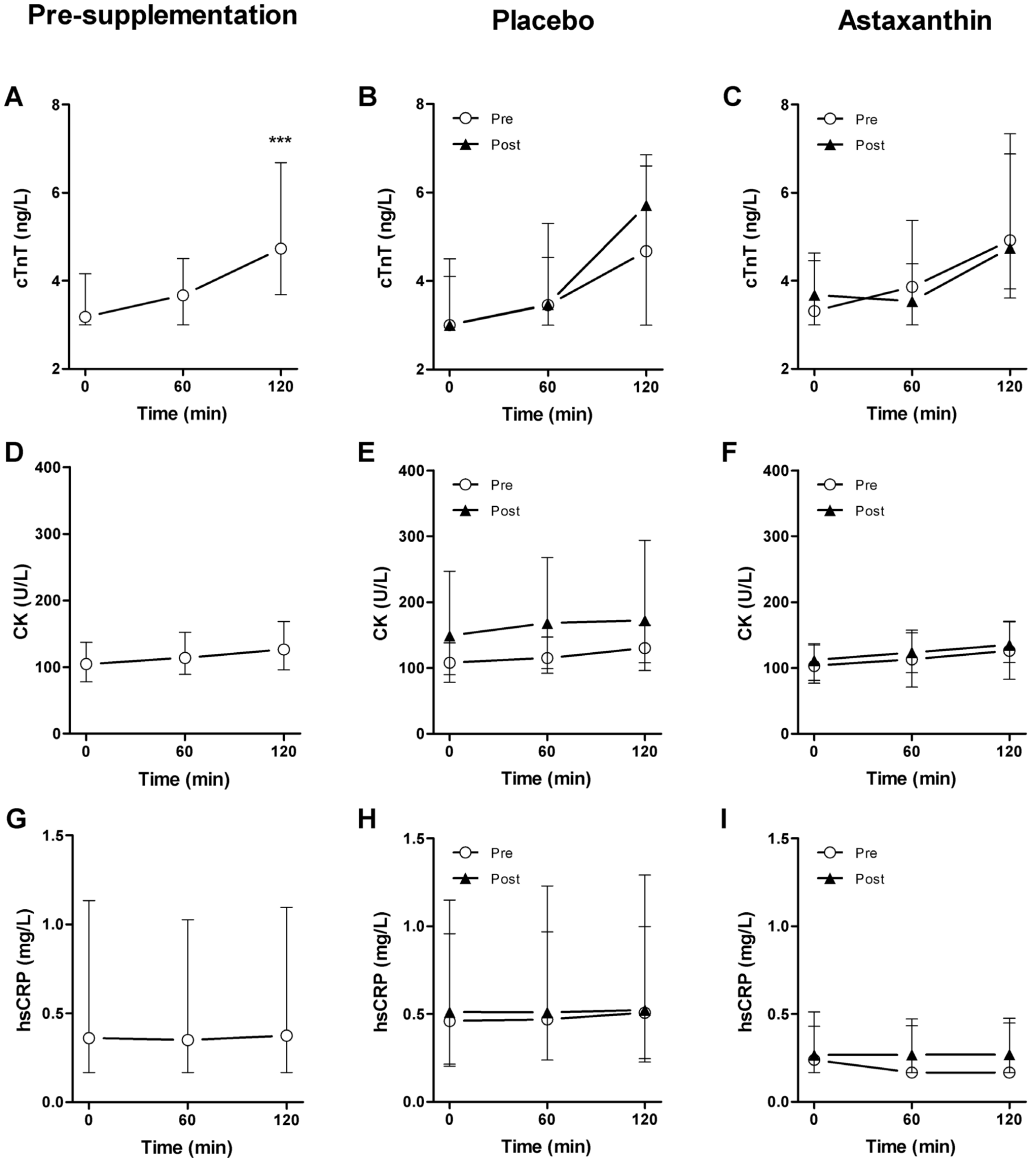
cTnT, CK and hsCRP concentrations during the pre- and post-supplementation exercise trials. A, D, G. Whole group cTnT, CK and hsCRP concentrations during the pre-supplementation exercise trials. B, E, H. cTnT, CK and hsCRP concentrations in the placebo group measured during the pre- and post-supplementation cycling trials. C, F, I. cTnT, CK and hsCRP concentrations in the astaxanthin group during the pre- and post-supplementation trials. ***denotes a significant increase from baseline (P<0.001). Values are median and interquartile range.

The pre-supplementation cycling trial induced a significant increase in cTnT, from overall median cTnT 3.2 (IQR 3.0–4.2) to 4.7 ng/L (IQR 3.7–6.7) immediately post-exercise (p<0.001) (Figure 2A). Four weeks of astaxanthin supplementation did not affect baseline cTnT concentrations compared to placebo supplementation (median 3.0 (IQR 3.0–4.5) versus 3.7 ng/L (IQR 3.0–4.6)) (p = 0.63). The post-supplementation cycling trial resulted in a significant increase from median cTnT 3.0 (IQR 3.0–4.5) to 5.7 ng/L (IQR 3.0–6.9) in the placebo group (Figure 2B), and from median cTnT 3.7 (IQR 3.0–4.7) to 4.8 ng/L (IQR 3.6–6.9) in the astaxanthin group, immediately post-exercise (Figure 2C) (both p<0.01). Despite the increase in basal astaxanthin concentrations, there were no differences between groups in the post-supplementation incremental area under the cTnT curve (p = 0.24). Furthermore, there was no significant association between the supplementation-induced astaxanthin concentration and exercise-induced cTn release (data not shown).

### Astaxanthin does not Improve Antioxidant Capacity in Highly Trained Individuals

Antioxidant capacity markers including trolox equivalent antioxidant capacity (TEAC), uric acid, and malondialdehyde (MDA) did not differ between the pre- and post-supplementation exercise trials or between groups. Markers of exercise-induced skeletal muscle damage (CK) (Figure 2, D–F) and inflammation (hsCRP) (Figure 2, G–I) were equally unaffected by astaxanthin supplementation. Furthermore, no evident relationship was detected between any of the antioxidant capacity markers and post-exercise cTnT concentrations (Figure S1).

## Discussion

In the present study we demonstrate that 4 weeks antioxidant supplementation did not improve antioxidant capacity, and did not reduce exercise-induced cTn release in well-trained individuals.

Intensive physical activity is characterized by an increase in oxygen consumption with concomitant production of reactive oxygen species. In the setting of AMI, generation of reactive oxygen species during the reintroduction of blood flow to a previously ischemic area, is a well established contributor to myocardial reperfusion injury and cTn release [35]. We hypothesized that a similar mechanism would apply in the setting of exercise-induced cTn release, and that oxidative stress associated with endurance exercise causes reversible or irreversible damage to cardiomyocytes with consequent release of cTnT.

The lack of any improvement in antioxidant capacity in the present study was unlikely due to non-compliance of the subjects. Compliance, estimated by pill-counting was >90% in 28 out of 31 subjects. Adequate compliance was also evident from strongly increased plasma astaxanthin concentrations. Furthermore, the effect of astaxanthin supplementation was not masked by differences in the total carotenoid intake during the experimental period or between groups. In previous studies, prolonged astaxanthin supplementation (12 weeks, 8 mg/day [36] or 12 weeks, 20 mg/day [37] improved total antioxidant capacity, attenuated MDA and lipid peroxidation. Possibly the highly trained state of our participants, which is associated with already substantially elevated endogenous antioxidant capacity [38], [39], precluded an additional improvement through antioxidant supplementation.

In line with the absence of any effect of astaxanthin on antioxidant capacity, no significant reduction of exercise-induced cTn release were observed in subjects following 4 weeks of antioxidant supplementation. To date, one previous study addressed the hypothesis that antioxidant supplementation (tart cherry juice) reduces exercise-induced cTn release in humans, and concluded that 5 days of antioxidant supplementation did not attenuate circulating cTn following a marathon run [13], [40]. Our study complements this work and deals with some limitations of the original report related to power, duration of antioxidant supplementation, and study design: we extended the period of antioxidant supplementation, increased the number of study subjects and applied a pre-post test design that allows each subject to act as its own control. Our approach circumvents power problems that may arise from the high between-person variation in exercise-induced cTn release [21], and exploits the good reproducibility and low within-person variation of cTn release during two identical exercise bouts [21].

In conclusion, despite substantial increases in plasma astaxanthin concentrations, astaxanthin supplementation did not improve antioxidant capacity in highly trained cyclists. Accordingly, exercise-induced cTnT concentrations were not affected by astaxanthin supplementation.

## Supporting Information

Figure S1 **Relation between exercise-induced cTnT concentrations and markers of antioxidant capacity.** Post-exercise cTnT concentrations during the post-supplementation trial are plotted against post-supplementation concentrations of TEAC (A), MDA (B) and Uric Acid (C).
(TIF)

Protocol S1 **Trial protocol.**
(DOC)

Checklist S1 **CONSORT checklist.**
(DOC)

## References

[1] Thygesen K, Alpert JS, Jaffe AS, Simoons ML, Chaitman BR, et al (2012) Third universal definition of myocardial infarction. Eur Heart J 33: 2551–2567. 22922414 10.1093/eurheartj/ehs184

[2] Agewall S, Giannitsis E, Jernberg T, Katus H (2011) Troponin elevation in coronary vs. non-coronary disease. Eur Heart J 32: 404–411. 21169615 10.1093/eurheartj/ehq456

[3] Kelley WE, Januzzi JL, Christenson RH (2009) Increases of cardiac troponin in conditions other than acute coronary syndrome and heart failure. Clin Chem 55: 2098–2112. 19815610 10.1373/clinchem.2009.130799

[4] Saunders JT, Nambi V, de Lemos JA, Chambless LE, Virani SS, et al (2011) Cardiac troponin T measured by a highly sensitive assay predicts coronary heart disease, heart failure, and mortality in the Atherosclerosis Risk in Communities Study. Circulation 123: 1367–1376. 21422391 10.1161/CIRCULATIONAHA.110.005264 PMC3072024

[5] van Wijk S, Jacobs L, Eurlings LW, van Kimmenade R, Lemmers R, et al (2012) Troponin T measurements by high-sensitivity vs conventional assays for risk stratification in acute dyspnea. Clin Chem 58: 284–292. 22100806 10.1373/clinchem.2011.175976

[6] Eggers KM, Venge P, Lindahl B, Lind L (2013) Cardiac troponin I levels measured with a high-sensitive assay increase over time and are strong predictors of mortality in an elderly population. J Am Coll Cardiol 61: 1906–1913. 23500239 10.1016/j.jacc.2012.12.048

[7] Omland T, Pfeffer MA, Solomon SD, de Lemos JA, Rosjo H, et al (2013) Prognostic value of cardiac troponin I measured with a highly sensitive assay in patients with stable coronary artery disease. J Am Coll Cardiol 61: 1240–1249. 23414791 10.1016/j.jacc.2012.12.026

[8] Neumayr G, Pfister R, Mitterbauer G, Maurer A, Gaenzer H, et al (2002) Effect of the “Race Across The Alps”. in elite cyclists on plasma cardiac troponins I and T. Am J Cardiol 89: 484–486. 10.1016/s0002-9149(01)02280-9 11835940

[9] Middleton N, George K, Whyte G, Gaze D, Collinson P, et al (2008) Cardiac troponin T release is stimulated by endurance exercise in healthy humans. J Am Coll Cardiol 52: 1813–1814. 19022162 10.1016/j.jacc.2008.03.069

[10] Eijsvogels T, George K, Shave R, Gaze D, Levine BD, et al (2010) Effect of prolonged walking on cardiac troponin levels. Am J Cardiol 105: 267–272. 20102930 10.1016/j.amjcard.2009.08.679

[11] Mingels A, Jacobs L, Michielsen E, Swaanenburg J, Wodzig W, et al (2009) Reference population and marathon runner sera assessed by highly sensitive cardiac troponin T and commercial cardiac troponin T and I assays. Clin Chem 55: 101–108. 18988757 10.1373/clinchem.2008.106427

[12] Neumayr G, Pfister R, Mitterbauer G, Eibl G, Hoertnagl H (2005) Effect of competitive marathon cycling on plasma N-terminal pro-brain natriuretic peptide and cardiac troponin T in healthy recreational cyclists. Am J Cardiol 96: 732–735. 16125505 10.1016/j.amjcard.2005.04.054

[13] Howatson G, Goodall S, Hill J, Brouner J, Gaze D, et al (2011) Antioxidant supplementation does not attenuate exercise-induced cardiac troponin release. Int J Cardiol 152: 101–102. 21813194 10.1016/j.ijcard.2011.07.006

[14] Scherr J, Braun S, Schuster T, Hartmann C, Moehlenkamp S, et al (2011) 72-h kinetics of high-sensitive troponin T and inflammatory markers after marathon. Med Sci Sports Exerc 43: 1819–1827. 21448080 10.1249/MSS.0b013e31821b12eb

[15] Katus HA, Remppis A, Scheffold T, Diederich KW, Kuebler W (1991) Intracellular compartmentation of cardiac troponin T and its release kinetics in patients with reperfused and nonreperfused myocardial infarction. Am J Cardiol 67: 1360–1367. 1904190 10.1016/0002-9149(91)90466-x

[16] Hickman PE, Potter JM, Aroney C, Koerbin G, Southcott E, et al (2010) Cardiac troponin may be released by ischemia alone, without necrosis. Clin Chim Acta 411: 318–323. 20036224 10.1016/j.cca.2009.12.009

[17] Shave R, Baggish A, George K, Wood M, Scharhag J, et al (2010) Exercise-induced cardiac troponin elevation: evidence, mechanisms, and implications. J Am Coll Cardiol 56: 169–176. 20620736 10.1016/j.jacc.2010.03.037

[18] Shave R, George KP, Atkinson G, Hart E, Middleton N, et al (2007) Exercise-induced cardiac troponin T release: a meta-analysis. Med Sci Sports Exerc 39: 2099–2106. 18046180 10.1249/mss.0b013e318153ff78

[19] Bergmann O, Bhardwaj RD, Bernard S, Zdunek S, Barnabe-Heider F, et al (2009) Evidence for cardiomyocyte renewal in humans. Science 324: 98–102. 19342590 10.1126/science.1164680 PMC2991140

[20] Mehta R, Gaze D, Mohan S, Williams KL, Sprung V, et al (2012) Post-exercise cardiac troponin release is related to exercise training history. Int J Sports Med 33: 333–337. 22377942 10.1055/s-0031-1301322

[21] Klinkenberg LJ, Res PT, van Loon LJ, van Dieijen-Visser MP, Meex SJ (2012) Strong link between basal and exercise-induced cardiac troponin T levels: do both reflect risk? Int J Cardiol 158: 129–131. 22560936 10.1016/j.ijcard.2012.04.050

[22] La Gerche A, Burns AT, Mooney DJ, Inder WJ, Taylor AJ, et al (2012) Exercise-induced right ventricular dysfunction and structural remodelling in endurance athletes. Eur Heart J 33: 998–1006. 22160404 10.1093/eurheartj/ehr397

[23] Heidbuchel H, Prior DL, La Gerche A (2012) Ventricular arrhythmias associated with long-term endurance sports: what is the evidence? Br J Sports Med 46 Suppl 1i44–i50. 23097479 10.1136/bjsports-2012-091162

[24] Heidbuchel H, Hoogsteen J, Fagard R, Vanhees L, Ector H, et al (2003) High prevalence of right ventricular involvement in endurance athletes with ventricular arrhythmias. Role of an electrophysiologic study in risk stratification. Eur Heart J 24: 1473–1480. 12919770 10.1016/s0195-668x(03)00282-3

[25] Wilson M, O’Hanlon R, Prasad S, Deighan A, Macmillan P, et al (2011) Diverse patterns of myocardial fibrosis in lifelong, veteran endurance athletes. J Appl Physiol 110: 1622–1626. 21330616 10.1152/japplphysiol.01280.2010 PMC3119133

[26] Nie J, Close G, George KP, Tong TK, Shi Q (2010) Temporal association of elevations in serum cardiac troponin T and myocardial oxidative stress after prolonged exercise in rats. Eur J Appl Physiol 110: 1299–1303. 20711602 10.1007/s00421-010-1604-6

[27] Whyte G, George K, Shave R, Dawson E, Stephenson C, et al (2005) Impact of marathon running on cardiac structure and function in recreational runners. Clin Sci (Lond) 108: 73–80. 15377277 10.1042/CS20040186

[28] Res PT, Cermak NM, Stinkens R, Tollakson TJ, Haenen GR, et al (2013) Astaxanthin supplementation does not augment fat use or improve endurance performance. Med Sci Sports Exerc 45: 1158–1165. 23274592 10.1249/MSS.0b013e31827fddc4

[29] Jeukendrup A, Saris WH, Brouns F, Kester AD (1996) A new validated endurance performance test. Med Sci Sports Exerc 28: 266–270. 8775164 10.1097/00005768-199602000-00017

[30] Aoi W, Naito Y, Sakuma K, Kuchide M, Tokuda H, et al (2003) Astaxanthin limits exercise-induced skeletal and cardiac muscle damage in mice. Antioxid Redox Signal 5: 139–144. 12626126 10.1089/152308603321223630

[31] Iwamoto T, Hosoda K, Hirano R, Kurata H, Matsumoto A, et al (2000) Inhibition of low-density lipoprotein oxidation by astaxanthin. J Atheroscler Thromb 7: 216–222. 11521685 10.5551/jat1994.7.216

[32] Giannitsis E, Kurz K, Hallermayer K, Jarausch J, Jaffe AS, et al (2010) Analytical validation of a high-sensitivity cardiac troponin T assay. Clin Chem 56: 254–261. 19959623 10.1373/clinchem.2009.132654

[33] Luden ND, Saunders MJ, Todd MK (2007) Postexercise carbohydrate-protein- antioxidant ingestion decreases plasma creatine kinase and muscle soreness. Int J Sport Nutr Exerc Metab 17: 109–123. 17460336 10.1123/ijsnem.17.1.109

[34] Zoppi CC, Hohl R, Silva FC, Lazarim FL, Neto JM, et al (2006) Vitamin C and e supplementation effects in professional soccer players under regular training. J Int Soc Sports Nutr 3: 37–44. 18500971 10.1186/1550-2783-3-2-37 PMC2129167

[35] Park JL, Lucchesi BR (1999) Mechanisms of myocardial reperfusion injury. Ann Thorac Surg 68: 1905–1912. 10585102 10.1016/s0003-4975(99)01073-5

[36] Karppi J, Rissanen TH, Nyyssonen K, Kaikkonen J, Olsson AG, et al (2007) Effects of astaxanthin supplementation on lipid peroxidation. Int J Vitam Nutr Res 77: 3–11. 17685090 10.1024/0300-9831.77.1.3

[37] Choi HD, Youn YK, Shin WG (2011) Positive effects of astaxanthin on lipid profiles and oxidative stress in overweight subjects. Plant Foods Hum Nutr 66: 363–369. 21964877 10.1007/s11130-011-0258-9

[38] Gomez-Cabrera MC, Domenech E, Vina J (2008) Moderate exercise is an antioxidant: upregulation of antioxidant genes by training. Free Radic Biol Med 44: 126–131. 18191748 10.1016/j.freeradbiomed.2007.02.001

[39] Radak Z, Chung HY, Goto S (2008) Systemic adaptation to oxidative challenge induced by regular exercise. Free Radic Biol Med 44: 153–159. 18191751 10.1016/j.freeradbiomed.2007.01.029

[40] Howatson G, McHugh MP, Hill JA, Brouner J, Jewell AP, et al (2010) Influence of tart cherry juice on indices of recovery following marathon running. Scand J Med Sci Sports 20: 843–852. 19883392 10.1111/j.1600-0838.2009.01005.x

