# Supplementary material for: Effect of Antioxidant Supplementation on Exercise-Induced Cardiac Troponin Release in Cyclists: A Randomized Trial
Source: PLoS One. 2013 Nov 19;8(11):e79280. doi: 10.1371/journal.pone.0079280 (PMC3834092; doi:10.1371/journal.pone.0079280)
Supplement: Protocol S1 — Trial protocol. (DOC) [file pone.0079280.s002.doc]

Astaxanthin supplementation to augment fat oxidation and improve performance in endurance trained athletes

Astaxanthin supplementation to augment fat oxidation and improve performance in endurance trained athletes

| **Protocol ID** | MEC |
| --- | --- |
| **Short title** | Astaxanthin supplementation in cyclists |
| **Version** | II (two) |
| **Date** | Saturday, 29 August 2026 |
| **Coordinating investigator/project leader** | Dr. L.J.C. van Loon  School for Nutrition, Toxicology and Metabolism (NUTRIM)  Department of Human Movement Sciences  Maastricht University Medical Centre+  Maastricht  P.O. Box 616  6200 MD Maastricht  L.vanLoon@maastrichtuniversity.nl  Tel. +31 43 388 1397  Fax. +31 43 367 0972 |
| **Principal investigator** | Ir. P.T. Res, PhD student  School for Nutrition, Toxicology and Metabolism (NUTRIM)  Department of Human Movement Sciences  Maastricht University Medical Centre+  Maastricht  P.O. Box 616  6200 MD Maastricht  Peter.Res@maastrichtuniversity.nl  Tel. +31 43 388 1390  Fax. +31 43 367 0972 |
| **Sponsor** | - |
| **Responsible physician** | Bart Groen, MD  Department of Human Movement Sciences  Tel 043-3881390 |
| **Independent physician(s)** | Prof. Dr. H. Kuipers, MD  Department of Human Movement Sciences  Tel: 043-3881493 |
| **Laboratory sites** | Not applicable |
| **Pharmacy** | Not applicable |

**PROTOCOL SIGNATURE SHEET**

| **Name** | **Signature** | **Date** |
| --- | --- | --- |
| **Sponsor or legal representative:**  **For non-commercial research,**  **Head of Department:**  *Dr. L. Van Loon* |  |  |
| **Coordinating Investigator/Project leader/Principal Investigator:**  *Ir P.T. Res* |  |  |

**TABLE OF CONTENTS**

1. INTRODUCTION AND RATIONALE [7](#__RefHeading___Toc270680802)

2. OBJECTIVES [7](#__RefHeading___Toc270680803)

3. STUDY DESIGN [7](#__RefHeading___Toc270680804)

3.1.1 Protocol [7](#__RefHeading___Toc270680805)

4. STUDY POPULATION [9](#__RefHeading___Toc270680806)

4.1 Population [9](#__RefHeading___Toc270680807)

4.2 Inclusion criteria [9](#__RefHeading___Toc270680808)

4.3 Exclusion criteria [9](#__RefHeading___Toc270680809)

4.4 Sample size calculation [10](#__RefHeading___Toc270680810)

5. TREATMENT OF SUBJECTS [10](#__RefHeading___Toc270680811)

5.1 Investigational product/treatment [10](#__RefHeading___Toc270680812)

5.1.1 Supplements [10](#__RefHeading___Toc270680813)

6. METHODS [10](#__RefHeading___Toc270680814)

6.1 Study parameters/endpoints [10](#__RefHeading___Toc270680815)

6.1.1 Main study parameter/endpoint [11](#__RefHeading___Toc270680816)

6.1.2 Secondary study parameters/endpoints [11](#__RefHeading___Toc270680817)

6.1.3 Other study parameters [11](#__RefHeading___Toc270680818)

6.2 Randomisation, blinding and treatment allocation [11](#__RefHeading___Toc270680819)

6.3 Study procedures [11](#__RefHeading___Toc270680820)

6.3.1 Pretesting [11](#__RefHeading___Toc270680821)

6.3.2 Diet and activity prior to test [11](#__RefHeading___Toc270680822)

6.3.3 Protocol [12](#__RefHeading___Toc270680823)

6.3.4 Analysis [12](#__RefHeading___Toc270680824)

6.4 Withdrawal of individual subjects [12](#__RefHeading___Toc270680825)

6.4.1 Specific criteria for withdrawal [12](#__RefHeading___Toc270680826)

6.5 Replacement of individual subjects after withdrawal [12](#__RefHeading___Toc270680827)

6.6 Follow-up of subjects withdrawn from treatment [12](#__RefHeading___Toc270680828)

6.7 Premature termination of the study [12](#__RefHeading___Toc270680829)

7. SAFETY REPORTING [12](#__RefHeading___Toc270680830)

7.1 Section 10 WMO event [12](#__RefHeading___Toc270680831)

7.2 Adverse and serious adverse events [12](#__RefHeading___Toc270680832)

7.2.1 Suspected unexpected serious adverse reactions (SUSAR) [13](#__RefHeading___Toc270680833)

7.2.2 Annual safety report [13](#__RefHeading___Toc270680834)

7.3 Follow-up of adverse events [13](#__RefHeading___Toc270680835)

7.4 Data Safety Monitoring Board (DSMB) [13](#__RefHeading___Toc270680836)

8. STATISTICAL ANALYSIS [13](#__RefHeading___Toc270680837)

8.1 Descriptive statistics [13](#__RefHeading___Toc270680838)

9. ETHICAL CONSIDERATIONS [13](#__RefHeading___Toc270680839)

9.1 Regulation statement [13](#__RefHeading___Toc270680840)

9.2 Recruitment and consent [13](#__RefHeading___Toc270680841)

9.3 Objection by minors or incapacitated subjects [14](#__RefHeading___Toc270680842)

9.4 Benefits and risks assessment, group relatedness [14](#__RefHeading___Toc270680843)

9.5 Compensation for injury [14](#__RefHeading___Toc270680844)

9.6 Incentives [14](#__RefHeading___Toc270680845)

10. ADMINISTRATIVE ASPECTS AND PUBLICATION [14](#__RefHeading___Toc270680846)

10.1 Handling and storage of data and documents [14](#__RefHeading___Toc270680847)

10.2 Amendments [14](#__RefHeading___Toc270680848)

10.3 Annual progress report [15](#__RefHeading___Toc270680849)

10.4 End of study report [15](#__RefHeading___Toc270680850)

10.5 Public disclosure and publication policy [15](#__RefHeading___Toc270680851)

11. REFERENCES [15](#__RefHeading___Toc270680852)

**Summary:** Astaxanthin has been shown to be a powerful anti-oxidant. Health benefits have been shown in animals and humans. Exercise enhancing effects have been shown in animals via enhancement of fat oxidation. We want to examine the effects of 4 weeks of astaxanthin supplementation on fat oxidation and cycling performance in trained cyclists.

**Rationale:** The aim of this proposal is to investigate the effect of 4 weeks supplementation with astaxanthin on fat oxidation during exercise and exercise performance.

**Objective**: To test the hypothesis that 4 weeks supplementation with astaxanthin will improve fat oxidation and improve exercise performance in trained cyclists.

**Study design:** Double blind, placebo controlled study.

**Study population:** Well trained cyclists between 18-30 years old.

**Intervention:** astaxanthin (20 mg/day) or placebo for 4 weeks

**Main study parameters/endpoints:** The primary endpoint will be performance on a time trial after 60 min steady state exercise. Secondary endpoint will be fat-oxidation during the 90 minutes steady state 60% W_max_ exercise.

**Nature and extent of the burden and risks associated with participation, benefit and group relatedness:** The risks involved in participating in this experiment are minimal. Astaxanthin has been shown to be safe. Insertion of the catheters in a vein is comparable to a normal blood draw and the only risk is of a small local haematoma.

# INTRODUCTION AND RATIONALE

The two major substrates of energy production are fat and carbohydrate. A mixture of the two substrates is being used by muscle tissue during exercise [1, 2]. The ratio between the use of fat and carbohydrate depends on exercise intensity, endogenous carbohydrate availability, training status, diet and genotype [3]. One of the main limiting factors for endurance exercise performance capacity is the availability of muscle glycogen. By enhancing fat oxidation in favor of glycogen oxidation, muscle glycogen can be spared. Consequently, stimulating fat oxidation is the main interventional strategy to improve endurance exercise performance.

Astaxanthin is a carotenoid with high anti-oxidant properties. Astaxanthin is a naturally occurring substance, synthesized by the microalgae *Haematococcus pluvialis*. Several clinical studies have found beneficial effects of astaxanthin in humans [4-7], which has been attributed to its anti-oxidant and anti-inflammatory properties. The effective doses in these human studies were 16 mg/day [4], 40 mg/day [5], 8 mg/day [6], and 12 and 18 mg/day[7]. Few studies have used different doses of astaxanthin. The studies that have used different doses in general found more effect in higher doses. Iwamoto et al. provided doses of 1.8, 3.6, 14.4 and 21.6 mag/day and the highest LDL-oxidation lag times were found in the two highest doses [8]. Yoshida et al. provided 0, 6, 12 and 18 mg/day and found the highest changes in adiponectin and triglycerides in two highest doses [7] Kupcikinas et al. found a significant reduction in reflux syndrome in 40 mg/day compared to 16 mg/day and 0 mg/day [5]. Furthermore, animal studies have shown promising results on endurance performance as well. Aoi et al. examined muscle damage after exercise in mice and found a protective effect of astaxanthin [9]. Ikeuchi et al. found a performance enhancing effect during a 5 week supplementation protocol. They measured performance every week and saw a gradual increase in performance over time and a dose response relationship, with the highest time to exhaustion found in the mice supplemented with the highest dose of astaxanthin [10]. In an attempt to explain the previous findings, Aoi et al. investigated lipid metabolism during exercise and carnitine palmityol transferase I (CPT I), a rate limiting step in fat oxidation. An increased fat oxidation during exercise was found. Furthermore, a possible mechanism to explain their findings was found. In the astaxanthin group, less modification of CPT I occurred. The authors concluded that astaxanthin protects CPT I from oxidative stress, and hence increases the capacity to transport fatty acids over the mitochondrial membrane, facilitating lipid oxidation.

As a consequence astaxanthin is now widely used in nutritional supplements to enhance exercise performance and also in nutritional supplements to support weight loss. However, so far the impact of astaxanthin supplementation on skeletal muscle substrate use and endurance type exercise performance has not been studied in humans. The present study aims to investigate the effects of 4 week astaxanthin supplementation on measures of performance and substrate utilization in healthy, well-trained endurance athletes.

# OBJECTIVES

Primary objective of this study is to test the hypothesis that 4 week supplementation of astaxanthin will improve exercise performance. Therefore, the primary endpoint will be performance on a time trial. Secondary endpoints are fat oxidation at rest and during submaximal exercise.

# STUDY DESIGN

### Protocol

An outline of the study and the exercise protocol is provided in Figure 1. Subjects will be randomized to a 4-wk supplementation protocol (astaxanthine or placebo) and an exercise performance test will be done before and after the 4-wk intervention. For the exercise performance test, subjects will report to the laboratory at 8.00 h. Resting metabolic rate will be measured by ventilated hood connected to the OmniCal IV for indirect calorimetry and substrate use measurement. Before testing, participants will be weighed, fitted with a heart-rate monitor, and familiarized with the Borg scale of perceived exertion [11]. A Teflon catheter will be inserted in an antecubital vein.

The exercise protocol consists of 60 minutes steady state exercise at 50% Wmax (as determined in trial 1, see paragraph 6.3: pretesting). Subsequently, a time-trial of about an hour will be conducted. This type of time-trial has been validated and used before in our lab[12], see for an overview of validation studies Currell and Jeukendrup [13]. Examples of coefficients of variation in similar trials are 1.1 [14], 0.7 [15] and 0.9 [16].

Subjects will be instructed to perform a set amount of work in the shortest time possible. Total work to be performed will be calculated according to the equation of Jeukendrup, Saris, Brouns, and Kester [17], adapted based on previous experiences [12] :

Total amount of work = 0.60 · Wmax · 3,600

where Wmax is the maximal workload capacity determined at Visit 1 and 3,600 is the duration in seconds (equivalent to 1 hr). The ergometer will be set in linear mode so that 60% Wmax will be obtained when the participants cycle at their preferred cadence, which had been determined during Visit 1. The ergometer will be connected to a computer that calculates and displays the total amount of work performed. The only information participants receive is the absolute amount of work performed and the percentage of work performed relative to the set amount of work. This information will be displayed on a computer screen in front of the ergometer. A fan will be placed 1 m behind each participant to provide cooling and air circulation during the trials. The rating of perceived exertion (RPE) will be recorded at the start and when the time trial is completed, and heart rate (Polar, Finland) will be recorded continuously throughout the test. During none of the time trials, interaction will occur between the participant and the investigators except for Borg-scale recording. No encouragement will be given to the participants, and they will be kept unaware of performance-related information such as exercise time, heart rate, and cycling cadence.

Breath gas analysis will be performed during the steady state exercise for 5 minutes before every blood draw (15, 30, 45, 60 min) by indirect calorimetry with an OmniCal IV. Water will be provided during exercise ad libitum. 8 Blood samples (8ml) will subsequently be taken from an antecubital vein at t=0, 15, 30, 45, 60 and directly after completion of the time-trial.

***
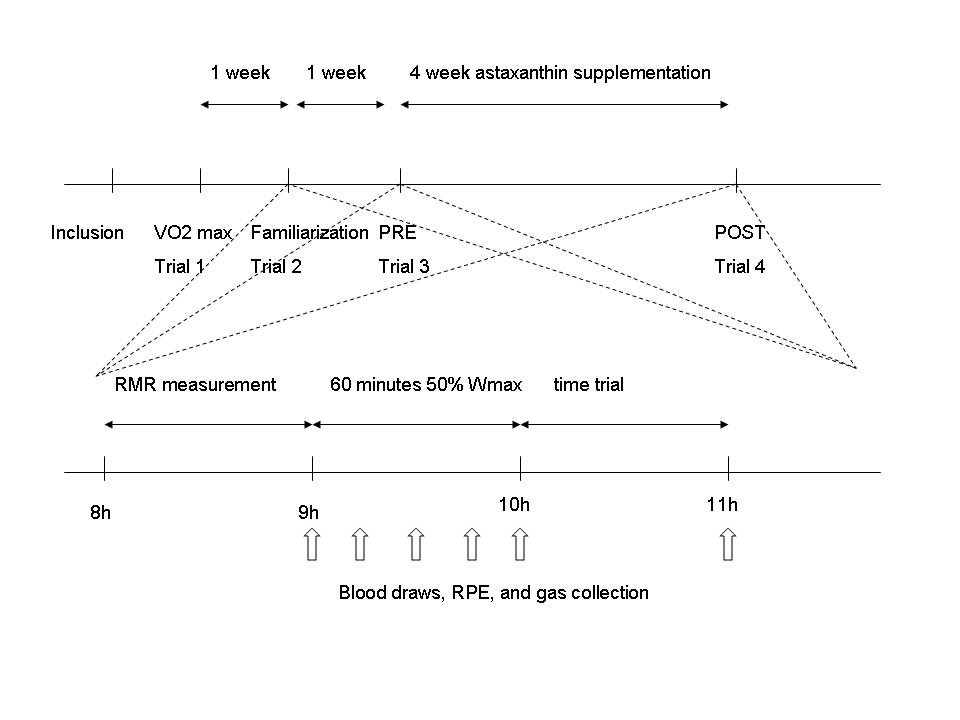
***

**Figure 1: Outline of study protocol.**

# STUDY POPULATION

## Population

We want to study trained, male cyclists to examine the effects of astaxanthin supplementation on performance and fat oxidation. All subjects have to be involved in regular cycling (competition), because this increases the reliability of a time trial performance [17] .We only include male athletes, because hormonal fluctuations in female athletes might influence their performance.

## Inclusion criteria

- Healthy
- Male
- Age between 18 and 30 years
- Endurance trained (≥3 sessions of endurance exercise per week)
- VO_2_ max ≥ 50 ml/kg/min
- Training history of more than one year of ≥3 sessions of endurance exercise per week
- BMI <25 kg/m^2^

## Exclusion criteria

- Use of medication
- Smoking

## Sample size calculation

A power calculation was performed with performance as primary outcome measure. With two independent groups, the sample size (N) was calculated with a power of 80% and a significance level of 0.05 with the following formula:

z _0.8_ = 0.84

z _0.975_ = 1.96

Based on previous data [12, 14-17], we expect the standard deviation of performance to be about 1.3% in both groups. An improvement of 60 seconds on the time trial is considered to be a worthwhile improvement, which corresponds to 1.7% .The calculated sample size then follows to be:

Taking into consideration a drop-out rate of 20%, the final number of subjects that should be recruited per group is 12. Since we include 2 groups the total amount of subjects that need to be recruited will be 24.

# TREATMENT OF SUBJECTS

## Investigational product/treatment

### Supplements

Subjects will be provided with either astaxanthin or placebo supplements. Subjects have to ingest 2 capsules of 4 mg with breakfast and 3 capsules with dinner throughout the 4 wk intervention period, to ascertain an intake of 20 mg/day. Astaxanthin has been shown to be safe in both animals [18] and humans [19, 20], and has recently received GRAS (generally recognized as safe) status by the US Food and Drug Administration [21]. This document also gives an excellent overview of all toxicological studies that have been performed (see attached file). Astaxanthin is produced by BioReal (Sweden). A certificate of safety for human consumption is attached.

The astaxanthin pills contain an algae extract high in astaxanthin, dissolved in sunflower oil in gelatine capsules (see attached file). The placebo treatment will consist of sunflower oil in gelatine capsules, without the astaxanthin. Treatments will look and taste identical. An independent person will randomly assign the subjects to a treatment so that both the subjects and the investigators involved in the study will be blinded to the treatment.

Astaxanthin or placebo pills have to be taken with meals (breakfast and dinner) twice daily. Astaxanthin and placebo pills will be send directly to the investigator. The supplements will be stored in a locked cabinet at room temperature, until the pills will be provided to the subjects. Compliance will be determined by the amount of pills that are left after the 4 week supplementation protocol.

# METHODS

## Study parameters/endpoints

The following parameters will be of interest for this study.

### Main study parameter/endpoint

Performance on the bicycle time trial will be the primary endpoint. The performance will be measured by the time it takes for the subject to complete a predetermined amount of work.;

### Secondary study parameters/endpoints

Fat oxidation at rest and during exercise. Blood measurements of glucose, lactate, free fatty acids, astaxanthin and cardiac troponin T.

### Other study parameters

Baseline values: VO_2_ max, W_max_, length, body weight, age, BMI.

## Randomisation, blinding and treatment allocation

Treatments will look and taste identical. An independent investigator will randomly assign the subjects to a treatment so that both the subjects and the investigators involved in the study will be blinded to the treatment. Randomization will be performed by a random number generator (www.random.org).

## Study procedures

### Pretesting

Body weight will be measured with a digital balance with an accuracy of 0.001 kg (E1200, August Sauter GmbH, Albstadt, Germany).

All subjects will participate in a cycling test (trial 1) to determine maximal oxygen uptake (VO_2_ max) and maximal workload capacity (Wmax). After a 5 min warm-up at 100 W, workload will be set at 150 W and will be increased with 50 W every 2.5 min until exhaustion [22]. O_2_ uptake and CO_2_ excretion, workload (W), cadence (rpm) and heart rate (Polar, Finland) will be recorded at every interval. The appropriate seat position, handle bar height and orientation will be determined and replicated in each subsequent visit. A familiarization trial (trial 2) will be performed to reduce learning effect over the pre and post exercise tests. The familiarization trial is identical to trials 3 and 4 (see paragraph 3.1: “protocol”)

### Diet and activity prior to test

Subjects will keep a 2-day food and physical activity diary. All subjects will be instructed to refrain from any sort of heavy physical labour. Subjects will receive a copy of their food diary and will be asked to replicate the food intake before subsequent visits. Additionally, all subjects will receive a standardized diet for the evening before the test, consisting of 55 En% carbohydrate, 15 En% protein and 30 En% fat. Subjects are allowed to eat ad libitum from the meal provided (frozen meal: Aviko familieschotel, 800g). The meal will be provided in insulated bags to keep the meal frozen during transport. Subjects will report fasted at the university for the performance tests. Subjects will be provided with a copy of their food diary. The nutritional intake the 2 days before trial 2 has to be replicated in the subsequent visits.

During the 4 week supplementation protocol, subjects are asked to refrain from eating salmon, shrimps, crab and lobster. Subjects will be asked to remain their habitual food intake and activity level during the 4 week intervention. Subjects are not allowed to take other supplements. If a subject does not comply to the guidelines, exclusion will be considered based on the type of supplement or severity of the violation of the guidelines.

### Protocol

An outline of the study protocol is provided at page 7 of this document and in figure 1 (page 9).

### Analysis

A total of 6 blood samples (8 ml) in trial 2, 3 and 4 will be collected in EDTA containing tubes and centrifuged at 1000 g and 4°C for 10 min. Aliquots of plasma will be frozen in liquid nitrogen and stored at –80°C until analysis. Glucose (Uni Kit III, Roche, Basel, Switzerland) and insulin will be analysed by radio immunoassay (Linco, Human Insulin RIA kit). Plasma free fatty acid concentrations will be analyzed with the NEFA C test kit from Wako Chemicals (Neuss, Germany). Astaxanthin content in plasma will be analyzed by reverse phase HPLC (Alliance 2690, Waters, Milford, MA). Breath gas analysis will be performed with an Omnical gas analyzer. Cardiac Troponin T will be measured by using the high-sensitivity cTnT assay on the Elecsys 2010 (Roche Diagnostics).

## Withdrawal of individual subjects

Subjects can leave the study at any time for any reason if they wish to do so without any consequences. The investigator can decide to withdraw a subject from the study for urgent medical reasons.

### Specific criteria for withdrawal

Not applicable.

## Replacement of individual subjects after withdrawal

In case of withdrawal, the subject will not be replaced. Power calculations are based on a drop-out rate of 20%.

## Follow-up of subjects withdrawn from treatment

Not applicable

## Premature termination of the study

Premature termination of the study does not need any form of explanation by the subject. Severe physical discomfort, noticed by the principal investigator, coordinating investigator or subjects themselves will lead to immediate termination of the study.

# SAFETY REPORTING

## Section 10 WMO event

In accordance to section 10, subsection 1, of the WMO, the investigator will inform the subjects and the reviewing accredited METC if anything occurs, on the basis of which it appears that the disadvantages of participation may be significantly greater than was foreseen in the research proposal. The study will be suspended pending further review by the accredited METC, except insofar as suspension would jeopardise the subjects’ health. The investigator will take care that all subjects are kept informed.

## Adverse and serious adverse events

Adverse events are defined as any undesirable experience occurring to a subject during a clinical trial, whether or not considered related to the investigational drug. All adverse events reported spontaneously by the subject or observed by the investiga­tor or his staff will be recorded.

A serious adverse event is any untoward medical occurrence or effect that at any dose results in death;

- is life threatening (at the time of the event);
- requires hospitalisation or prolongation of existing inpatients’ hospitalisation;
- results in persistent or significant disability or incapacity;
- is a congenital anomaly or birth defect;
- is a new event of the trial likely to affect the safety of the subjects, such as an unexpected outcome of an adverse reaction, lack of efficacy of an IMP used for the treatment of a life threatening disease, major safety finding from a newly completed animal study, etc.

All SAEs will be reported to the accredited METC that approved the protocol, according to the requirements of that METC.

### Suspected unexpected serious adverse reactions (SUSAR)

Based on previous comparable studies no SUSARs are expected (see safety issue addresses under “5.1.1 supplements”

### Annual safety report

Not applicable.

## Follow-up of adverse events

All adverse events will be followed until they have abated, or until a stable situation has been reached. Depending on the event, follow up may require additional tests or medical procedures as indicated, and/or referral to the general physician or a medical specialist.

## Data Safety Monitoring Board (DSMB)

Not applicable.

# STATISTICAL ANALYSIS

## Descriptive statistics

All data will be presented as means ± SEM. Statistical analysis of the data of performance will be performed using a repeated measures anova with time (pre-post) and treatment (astaxanthin vs placebo) as factors. Likewise, differences between treatments in plasma insulin, plasma glucose, plasma FFA, plasma astaxanthine, and whole-body fat oxidation following the intervention will be analysed with 2-way repeated measures ANOVA, with time as within-subjects factor, and treatment as between-subjects factor. Statistical significance will be set at P<0.05. Data will be analyzed with SPSS 15.0 (SPSS inc, USA).

# ETHICAL CONSIDERATIONS

## Regulation statement

This study will be conducted according to the principles of the declaration of Helsinki (version 9, October 2008) and in accordance with the Medical Research Involving Human Subjects Act (WMO)

## Recruitment and consent

All subjects will be recruited through local cycling clubs, advertisements in local newspapers and via posters at the MUMC^+^. In order to ascertain that the participants in this study are well informed, a written and oral explanation of all procedures of this study will be provided before their informed consent. After explaining all details, subjects will have 4 weeks to decide whether they want to participate. An independent MD (Prof. Dr. Harm Kuipers, tel: 043-388 1493) will be assigned to this project.

## Objection by minors or incapacitated subjects

Not applicable.

## Benefits and risks assessment, group relatedness

There are no benefits for the subjects in participating in the study, except for a possible performance enhancing effect of the astaxanthin supplementation. The risks involved in participating in this experiment are minimal. Astaxanthin has been shown to be safe. Insertion of the catheters in a vein is comparable to a normal blood draw and the only risk is of a small local haematoma.

## Compensation for injury

The sponsor/investigator has a liability insurance which is in accordance with article 7, subsection 6 of the WMO.

The sponsor (also) has an insurance which is in accordance with the legal requirements in the Netherlands (Article 7 WMO and the Measure regarding Compulsory Insurance for Clinical Research in Humans of 23th June 2003). This insurance provides cover for damage to research subjects through injury or death caused by the study.

1. € 450.000,-- (i.e. four hundred and fifty thousand Euro) for death or injury for each subject who participates in the Research;
2. € 3.500.000,-- (i.e. three million five hundred thousand Euro) for death or injury for all subjects who participate in the Research;
3. € 5.000.000,-- (i.e. five million Euro) for the total damage incurred by the organisation for all damage disclosed by scientific research for the Sponsor as ‘verrichter’ in the meaning of said Act in each year of insurance coverage.

The insurance applies to the damage that becomes apparent during the study or within 4 years after the end of the study.

## Incentives

Subjects will receive € 100,- for completion of the study. The latter also includes travel expenses*.* If the subject will not complete the study, he will receive payment according to ratio of the study completed.

# ADMINISTRATIVE ASPECTS AND PUBLICATION

## Handling and storage of data and documents

To protect the privacy of the participants, all collected data will be encoded and only P. Res, and L. van Loon will have access to the encoding key and the acquired data. All subjects will be offered full access to their own data and the outcomes of the project will be explained to them. All human material and subject data will be kept for a maximum of 15 years, after which all material will be destroyed.

## Amendments

All amendments to the protocol (i.e. regarding the intervention itself as well as the different measurements and/or tests performed) will be notified to the METC. Substantial amendments will be submitted for approval before they will be implemented in the study protocol. Annual reports will be submitted to the METC regarding progress of the study, number of subjects included/completed, and serious adverse events. Upon ending of the study, the METC will be notified within 8 weeks.

## Annual progress report

The investigator will submit a summary of the progress of the trial to the accredited METC once a year. Information will be provided on the date of inclusion of the first subject, numbers of subjects included and numbers of subjects that have completed the trial, serious adverse events/ serious adverse reactions, other problems, and amendments.

## End of study report

The sponsor will notify the accredited METC and the competent authority of the end of the study within a period of 90 days. The end of the study is defined as the last patient’s last visit.

In case the study is ended prematurely, the sponsor will notify the accredited METC and the competent authority within 15 days, including the reasons for the premature termination.

Within one year after the end of the study, the investigator/sponsor will submit a final study report with the results of the study, including any publications/abstracts of the study, to the accredited METC and the Competent Authority.

Estimated time scheduled:

Start: November 1^st^ 2010 (start immediately following approval MEC)

End: March 1^st^ 2011

## Public disclosure and publication policy

The results of this investigation will be published in a high-impact, scientific journal, regardless of the outcome of this study. All subjects will be given insight into the (individual) results of this study.

# REFERENCES

List of abbreviations:

BMI body mass index

CPT-I carnitine palmityol transferase I

FFA free fatty acids

LDL low density lipoproteins

POST post supplementation

PRE pre-supplementation

RMR resting metabolic rate

RPE rating of perceived exertion

RPM revolutions per minute

VO_2_ (max) (maximal) oxygen uptake

W (max) (maximal) workload
